# Supplementary material for: Pots vs trammel nets: a catch comparison study in a Mediterranean small-scale fishery
Source: PeerJ. 2020 Jul 17;8:e9287. doi: 10.7717/peerj.9287 (PMC7370935; doi:10.7717/peerj.9287)
Supplement: Supplemental Information 1 — SE: Standard Error; GTR: Trammel nets; LP: large pots; SP: small pots. [file peerj-08-9287-s001.docx]

**Supplementary Table 1. Average catch per unit effort of the landed species, standardized in weight (CPUE_W_), for the three gears in the three sites. The most important species commented in the text are highlighted in bold. SE: Standard Error; GTR: Trammel nets; LP: large pots; SP: small pots.**

|  | **Marina di Ravenna** | | | **Senigallia** | | | **Portonovo** | |
| --- | --- | --- | --- | --- | --- | --- | --- | --- |
| **Species** | **GTR CPUE***_W_* **(mean ± SE)** | **LP CPUE*_W_* (mean ± SE)** | **SP CPUE*_W_* (mean ± SE)** | **GTR CPUE*_W_* (mean ± SE)** | **LP CPUE*_W_* (mean ± SE)** | **SP CPUE*_W_* (mean ± SE)** | **GTR CPUE*_W_* (mean ± SE)** | **SP CPUE*_W_* (mean ± SE)** |
| **FISHES** |  |  |  |  |  |  |  |  |
| *Boops boops* | **-** | 0.017 ± 0.001 | **-** | **-** | - | - | **-** | **-** |
| *Chelidonichthys lucernus* | - | 0.001 ± 0.001 | - | 0.024 ± 0.010 | - | - | - | - |
| ***Conger conger*** | **-** | **0.462 ± 0.262** | **0.097 ± 0.097** | **-** | **-** | **-** | **-** | **-** |
| ***Dentex dentex*** | **-** | **-** | **-** | **-** | **-** | **-** | **-** | **0.328 ± 0.328** |
| ***Diplodus annularis*** | **0.118 ± 0.118** | **0.356 ± 0.136** | **0.126 ± 0.054** | **0.569 ± 0.138** | **0.328 ± 0.131** | **0.730 ± 0.195** | **-** | **-** |
| *Diplodus vulgaris* | 0.019 ± 0.019 | 0.007 ± 0.004 | - | - | - | - | - | 0.091 ± 0.091 |
| *Gobius niger* | - | - | - | 0.076 ± 0.037 | 0.023 ± 0.015 | 0.045 ± 0.020 | 0.028 ± 0.028 | - |
| *Gobius paganellus* | - | 0.001 ± 0.001 | 0.002 ± 0.002 | - | - | - | - | - |
| ***Lithognathus mormyrus*** | **-** | **-** | **0.017 ± 0.013** | **0.067 ± 0.053** | **0.383 ± 0.246** | **0.195 ± 0.132** | **0.053 ± 0.036** | **0.359 ± 0.246** |
| ***Liza aurata*** | **-** | **-** | **-** | **0.543 ± 0.362** | **-** | **-** | **-** | **-** |
| *Merlangius merlangus* | - | 0.001 ± 0.001 | - | - | - | - | - | - |
| ***Mugil cephalus*** | **-** | **0.028 ± 0.019** | **0.013 ± 0.013** | **-** | **-** | **-** | **0.304 ± 0.125** | **-** |
| *Mullus surmuletus* | 0.008 ± 0.008 | 0.005 ± 0.005 | 0.007 ± 0.007 | - | - | - | - | - |
| *Oblada melanura* | - | 0.010 ± 0.010 | - | - | - | - | - | - |
| *Pagellus erythrinus* | - | - | - | 0.021 ± 0.016 | - | - | - | - |
| *Raja asterias* | 0.016 ± 0.016 | - | - | - | - | - | - | - |
| ***Sarda sarda*** | **-** | **-** | **-** | **0.452 ± 0.452** | **-** | **-** | **-** | **-** |
| ***Sciaena umbra*** | **0.316 ± 0.179** | **2.384 ± 1.547** | **0.488 ± 0.209** | **0.081 ± 0.081** | **-** | **-** | **-** | **0.011 ± 0.007** |
| *Scomber scombrus* | - | - | - | 0.088 ± 0.059 | - | - | - | - |
| ***Scophthalmus rhombus*** | **-** | **-** | **-** | **0.252 ± 0.252** | **-** | **-** | **-** | **-** |
| *Scorpaena notata* | - | - | - | 0.014 ± 0.014 | - | - | - | - |
| *Scorpaena porcus* | - | - | - | - | - | - | 0.138 ± 0.058 | 0.006 ± 0.006 |
| *Scorpaena scrofa* | - | - | - | - | - | - | 0.024 ± 0.017 | 0.015 ± 0.015 |
| *Solea impar* | - | - | - | 0.011 ± 0.011 | - | - | - | - |
| *Solea lascaris* | 0.175 ± 0.121 | - | - | - | - | - | - | - |
| ***Solea solea*** | **0.792 ± 0.455** | **0.012 ± 0.010** | **0.021 ± 0.012** | **0.445 ± 0.188** | **-** | **-** | **-** | **0.011 ± 0.011** |
| *Sparus aurata* | - | - | - | 0.024 ± 0.024 | - | - | - | 0.005 ± 0.005 |
| *Sphyraena sphyraena* | - | - | - | 0.157 ± 0.082 | - | - | - | - |
| *Trachurus mediterraneus* | - | - | - | - | - | - | 0.014 ± 0.014 | - |
| *Trachurus spp.* | - | - | - | 0.015 ± 0.015 | - | 0.019 ± 0.019 | - | - |
| *Umbrina cirrosa* | 0.154 ± 0.154 | - | - | 0.105 ± 0.079 | - | 0.006 ± 0.006 | 0.030 ± 0.030 | - |
| **CRUSTACEANS** |  |  |  |  |  |  |  |  |
| *Homarus gammarus* | 0.013 ± 0.013 | - | - | - | - | 0.041 ± 0.041 | - | - |
| *Maja crispata* | - | - | - | - | - | 0.004 ± 0.004 | - | - |
| *Maja squinado* | - | - | - | 0.222 ± 0.222 | - | - | - | - |
| *Melicertus kerathurus* | - | - | - | 0.120 ± 0.041 | - | - | - | 0.008 ± 0.008 |
| ***Squilla mantis*** | **0.447 ± 0.181** | **-** | **0.001 ± 0.001** | **0.033 ± 0.024** | **-** | **-** | **-** | **-** |
| **MOLLUSCS** |  |  |  |  |  |  |  |  |
| *Octopus vulgaris* | - | - | - | - | - | 0.116 ± 0.116 | - | 0.091 ± 0.091 |
| ***Sepia officinalis*** | **1.999 ± 0.518** | **4.007 ± 0.952** | **2.962 ± 0.562** | **1.024 ± 0.572** | **0.951 ± 0.570** | **1.931 ± 0.343** | **2.069 ± 0.552** | **1.558 ± 0.371** |
