# Supplementary material for: Pots vs trammel nets: a catch comparison study in a Mediterranean small-scale fishery
Source: PeerJ. 2020 Jul 17;8:e9287. doi: 10.7717/peerj.9287 (PMC7370935; doi:10.7717/peerj.9287)
Supplement: Supplemental Information 2 — SE: Standard Error; GTR: Trammel nets; LP: large pots; SP: small pots. [file peerj-08-9287-s002.docx]

**Supplementary Table 2. Average CPUE_W_, standardized in weight, of the discarded species caught by the three gears in the three sites. The most important species commented in the text are highlighted in bold. SE: Standard Error; GTR: Trammel nets; LP: large pots; SP: small pots.**

|  | **Marina di Ravenna** | | | **Senigallia** | | | **Portonovo** | |
| --- | --- | --- | --- | --- | --- | --- | --- | --- |
| **Species** | **GTR CPUE*_W_***  **(mean ± SE)** | **LP CPUE*_W_* (mean ± SE)** | **SP CPUE*_W_*  (mean ± SE)** | **GTR CPUE*_W_* (mean ± SE)** | **LP CPUE*_W_* (mean ± SE)** | **SP CPUE*_W_*  (mean ± SE)** | **GTR CPUE*_W_* (mean ± SE)** | **SP CPUE*_W_* (mean ± SE)** |
| **FISHES** |  |  |  |  |  |  |  |  |
| ***Alosa fallax*** | **0.240 ± 0.143** | **-** | **-** | **0.577 ± 0.473** | **-** | **-** | **-** | **-** |
| *Blennius ocellaris* | - | - | - | - | **-** | **-** | 0.007 ± 0.007 | - |
| *Boops boops* | - | 0.011 ± 0.008 | - | 0.026 ± 0.018 | - | - | - | - |
| *Chelidonichthys lucernus* | 0.034 ± 0.021 | 0.001 ± 0.001 | - | 0.048 ± 0.033 | - | - | - | - |
| *Engraulis encrasicolus* | - | - | - | 0.051 ± 0.051 | - | - | - | - |
| *Gobius paganellus* | - | 0.001 ± 0.001 | 0.009 ± 0.007 | - | - | - | - | - |
| *Liza aurata* | 0.031 ± 0.031 | - | - | - | - | - | - | - |
| *Merlangius merlangus* | 0.048 ± 0.024 | - | - | - | - | - | - | - |
| ***Pteroplatytrygon violacea*** | **0.184 ± 0.088** | **-** | **-** | **-** | **-** | **-** | **-** | **-** |
| *Raja asterias* | - | - | - | 0.026 ± 0.024 | - | - | - | - |
| *Sardina pilchardus* | - | - | - | 0.019 ± 0.013 | - | - | - | - |
| *Sardinella aurita* | 0.013 ± 0.013 | - | - | - | - | - | - | - |
| *Sciaena umbra* | - | 0.012 ± 0.012 | 0.008 ± 0.008 | - | - | - | - | - |
| *Scomber japonicus* | 0.019 ± 0.019 | - | - | - | - | - | - | - |
| *Solea solea* | 0.006 ± 0.006 | 0.002 ± 0.002 | - | - | - | - | - | - |
| *Sparus aurata* | - | - | - | - | - | - | - | 0.005 ± 0.005 |
| *Symphodus tinca* | - | - | - | - | - | - | 0.067 ± 0.067 | - |
| *Umbrina cirrosa* | - | - | - | 0.015 ± 0.015 | - | - | - | - |
| **CRUSTACEANS** |  |  |  |  |  |  |  |  |
| ***Eriphia verrucosa*** | **-** | **-** | **-** | **-** | **-** | **-** | **0.137 ± 0.090** | **-** |
| *Homarus gammarus* | 0.011 ± 0.011 | - | - | - | - | - | 0.013 ± 0.013 | - |
| *Liocarcinus depurator* | 0.068 ± 0.038 | - | - | - | - | - | 0.019 ± 0.019 | - |
| ***Liocarcinus vernalis*** | **0.051 ± 0.032** | **-** | **-** | **0.109 ± 0.089** | **-** | **-** | **0.009 ± 0.009** | **-** |
| ***Maja crispata*** | **-** | **-** | **-** | **-** | **-** | **0.004 ± 0.004** | **0.173 ± 0.112** | **-** |
| *Squilla mantis* | 0.036 ± 0.020 | - | 0.001 ± 0.001 | - | - | - | - | - |
| **MOLLUSCS** |  |  |  |  |  |  |  |  |
| ***Hexaplex trunculus*** | **-** | **-** | **-** | **-** | **-** | **-** | **0.101 ± 0.082** | **-** |
| *Mytilus galloprovincialis* | - | - | - | - | - | - | 0.078 ± 0.060 | - |
| *Nassarius mutabilis* | - | - | - | - | - | - | 0.016 ± 0.016 | - |
| *Ostrea edulis* | - | - | - | - | - | - | 0.014 ± 0.014 | - |
