# Supplementary material for: Pots vs trammel nets: a catch comparison study in a Mediterranean small-scale fishery
Source: PeerJ. 2020 Jul 17;8:e9287. doi: 10.7717/peerj.9287 (PMC7370935; doi:10.7717/peerj.9287)
Supplement: Supplemental Information 3 — GTR: Trammel nets; LP: large pots; SP: small pots. [file peerj-08-9287-s003.docx]

**Supplementary Table 3. Total CPUE_W_, standardized in weight (kg), of each haul for each gear and each site. GTR: Trammel nets; LP: large pots; SP: small pots.**

| **Area** | **GTR** | |  | **SP** | |  | **LP** | |
| --- | --- | --- | --- | --- | --- | --- | --- | --- |
|  | **ID Haul** | **CPUE_W_** |  | **ID Haul** | **CPUE_W_** |  | **ID Haul** | **CPUE_W_** |
| **Marina di Ravenna** | 150_GTR | 0 |  | 148_SP | 1.42 |  | 149_LP | 4.49 |
|  | 153_GTR | 7.34 |  | 151_SP | 5.22 |  | 152_LP | 15.82 |
|  | 156_GTR | 6.51 |  | 154_SP | 0.35 |  | 155_LP | 0.46 |
|  | 159_GTR | 5.17 |  | 158_SP | 6.29 |  | 157_LP | 8.11 |
|  | 162_GTR | 7.01 |  | 161_SP | 5.57 |  | 160_LP | 8.7 |
|  | 165_GTR | 10.98 |  | 164_SP | 9 |  | 163_LP | 8.24 |
|  | 168_GTR | 0 |  | 167_SP | 2.81 |  | 166_LP | 4.03 |
|  | 171_GTR | 3.23 |  | 170_SP | 6.55 |  | 169_LP | 11.66 |
|  | 174_GTR | 0.67 |  | 173_SP | 4.32 |  | 172_LP | 0.96 |
|  | 177_GTR | 7.88 |  | 176_SP | 8.3 |  | 175_LP | 11.95 |
|  | 180_GTR | 5.24 |  | 179_SP | 3.72 |  | 178_LP | 1.08 |
|  | 183_GTR | 0 |  | 182_SP | 1.7 |  | 181_LP | 1.74 |
|  | 186_GTR | 4.73 |  | 185_SP | 1.39 |  | 184_LP | 4.89 |
|  | 189_GTR | 8.72 |  | 188_SP | 0.09 |  | 187_LP | 10.72 |
|  | 192_GTR | 7.34 |  | 191_SP | 8.46 |  | 190_LP | 7.26 |
|  | 195_GTR | 0 |  | 194_SP | 2.52 |  | 193_LP | 5.4 |
|  | 198_GTR | 2.88 |  | 197_SP | 1.17 |  | 196_LP | 1.99 |
|  | 201_GTR | 1.8 |  | 200_SP | 1.83 |  | 199_LP | 2.33 |
|  | 204_GTR | 0 |  | 203_SP | 2.96 |  | 202_LP | 35.07 |
|  | 207_GTR | 1.63 |  | 206_SP | 0.74 |  | 205_LP | 0.5 |
|  |  |  |  |  |  |  |  |  |
| **Portonovo** | 104_GTR | 7.39 |  | 105_SP | 3.23 |  | - | - |
|  | 107_GTR | 3.79 |  | 106_SP | 0.59 |  | - | - |
|  | 109_GTR | 3.85 |  | 108_SP | 5.81 |  | - | - |
|  | 111_GTR | 4.76 |  | 110_SP | 0.72 |  | - | - |
|  | 112_GTR | 0.8 |  | 113_SP | 1.29 |  | - | - |
|  | 114_GTR | 0.62 |  | 115_SP | 1.53 |  | - | - |
|  | 116_GTR | 2.62 |  | 117_SP | 5.54 |  | - | - |
|  | 119_GTR | 0.53 |  | 118_SP | 1.03 |  | - | - |
|  | 120_GTR | 1.59 |  | 121_SP | 5.33 |  | - | - |
|  | 122_GTR | 5.36 |  | 123_SP | 1.7 |  | - | - |
|  | 132_GTR | 0.63 |  | 133_SP | 2.53 |  | - | - |
|  | 134_GTR | 0 |  | 135_SP | 0.41 |  | - | - |
|  |  |  |  |  |  |  |  |  |
| **Senigallia** | 57_GTR | 8.19 |  | 61_SP | 2.02 |  | 60_LP | 1.47 |
|  | 63_GTR | 0.34 |  | 65_SP | 3.38 |  | 129_LP | 0 |
|  | 66_GTR | 1.83 |  | 70_SP | 5.12 |  | 68_LP | 0.17 |
|  | 72_GTR | 6.41 |  | 74_SP | 1.88 |  | 130_LP | 0 |
|  | 76_GTR | 11.65 |  | 79_SP | 1.26 |  | 78_LP | 0.32 |
|  | 84_GTR | 2.4 |  | 82_SP | 2.88 |  | 80_LP | 0.43 |
|  | 86_GTR | 4.08 |  | 87_SP | 2.36 |  | 89_LP | 4.07 |
|  | 90_GTR | 1.54 |  | 94_SP | 5.85 |  | 93_LP | 2.08 |
|  | 95_GTR | 4.77 |  | 98_SP | 3.07 |  | 131_LP | 0 |
|  | 99_GTR | 2.2 |  | 103_SP | 3 |  | 102_LP | 8.31 |
